# Assessment of Inflammatory Scores in Severity Prediction for Elderly Patients with Odontogenic Infections

**DOI:** 10.3390/dj12110371

**Published:** 2024-11-20

**Authors:** Gianina Tapalaga, Luminita Maria Nica, Laura-Elena Cirligeriu, Bogdan Andrei Bumbu, Marius Pricop

**Affiliations:** 1 Department of Odontotherapy and Endodontics, Faculty of Dental Medicine, “Victor Babes” University of Medicine and Pharmacy Timisoara, Eftimie Murgu Square 2, 300041 Timisoara, Romania; tapalaga.gianina@umft.ro; 2 Department of Restorative Dentistry and Endodontics, Research Center TADERP, Faculty of Dental Medicine, “Victor Babes” University of Medicine and Pharmacy Timisoara, Eftimie Murgu Square 2, 300041 Timisoara, Romania; nica.luminita@umft.ro (L.M.N.); cirligeriu.laura@umft.ro (L.-E.C.); 3 Department of Dental Medicine, Faculty of Medicine and Pharmacy, University of Oradea, 410073 Oradea, Romania; 4 Discipline of Oral and Maxillo-Facial Surgery, Faculty of Dental Medicine, “Victor Babes” University of Medicine and Pharmacy Timisoara, Eftimie Murgu Square 2, 300041 Timisoara, Romania; pricop.marius@umft.ro

**Keywords:** prediction statistics, odontogenic infections, negative outcomes, elderly

## Abstract

**Background and Objectives:** Odontogenic infections (OIs) can lead to severe complications, especially in elderly patients due to age-related physiological changes and comorbidities. This study aims to evaluate the predictive accuracy of inflammatory scores—APACHE II, CURB-65, SOFA, and NEWS2—in determining the severity of odontogenic infections among elderly patients (>70 years) compared to younger patients (<70 years). **Materials and Methods:** A retrospective cohort study was conducted on patients diagnosed with an OI at the Maxillofacial Surgery Department between January 2018 and January 2024. Patients were divided into two groups: elderly patients (>70 years, n = 49) and younger patients (<70 years, n = 64). The Symptom Severity score (SS) of odontogenic infections was calculated for all patients. Inflammatory scores—APACHE II, CURB-65, SOFA, and NEWS2—were assessed at admission and correlated with infection severity. Additional subgroup analyses were performed based on comorbidities and infection sites. **Results:** Elderly patients exhibited significantly higher SS scores (mean 12.47 ± 2.93) compared to younger patients (mean 7.82 ± 2.17, *p* < 0.001). APACHE II, CURB-65, SOFA and NEWS2 scores were significantly elevated in the elderly group (all *p* < 0.001). The SOFA score demonstrated the highest predictive accuracy for severe OIs in elderly patients, with an area under the curve (AUC) of 0.89 (95% CI: 0.82–0.95). Subgroup analyses revealed that comorbidities such as diabetes mellitus and cardiovascular disease significantly influenced infection severity (*p* < 0.05). **Conclusions:** Inflammatory scores, particularly SOFA, are effective in predicting the severity of odontogenic infections in elderly patients. The integration of these scores into clinical practice may enhance early identification of high-risk patients and improve management strategies.

## 1. Introduction

Odontogenic infections (OI) remain a significant clinical concern due to their potential to cause severe complications if they are not promptly and effectively managed [1,2]. Despite advances in dental care and antimicrobial therapies, OIs can progress rapidly, leading to life-threatening conditions such as deep neck space infections and sepsis [3,4]. These infections originate from dental structures and can extend to adjacent tissues, necessitating timely intervention to prevent morbidity and mortality [5].

The elderly population is particularly vulnerable to severe OI outcomes [6,7]. Age-related physiological changes, a higher prevalence of comorbidities and immunosenescence contribute to an increased risk of infection severity and complications in older adults [8,9]. Moreover, delayed presentation and atypical symptomatology often complicate the timely diagnosis and treatment in this age group. Factors such as reduced pain perception, cognitive impairment and social barriers can lead to advanced disease stages upon presentation [10,11].

Accurate and early prediction of infection severity is crucial for optimizing patient outcomes. Clinical scoring systems such as the Symptom Severity score (SS) for odontogenic infections have been utilized to assess the extent and progression of the disease [12]. The SS score incorporates clinical signs, symptoms and laboratory parameters to stratify patients based on infection severity [13]. However, the applicability and predictive value of SS in elderly patients require further investigation, given their unique physiological characteristics.

In recent years, systemic inflammatory response markers and scoring systems have been explored as potential tools for predicting the severity of infections. Scores like APACHE II (Acute Physiology and Chronic Health Evaluation II) [14], CURB-65 [15], SOFA (Sequential Organ Failure Assessment) [16], and NEWS2 (National Early Warning Score 2) [17], initially developed for other clinical settings, have shown promise in assessing the severity of systemic infections and guiding clinical decision-making. These scores evaluate various physiological parameters and organ functions, providing an objective measure of patient status.

Previous studies have evaluated the role of these scores in various infections, including pneumonia and sepsis, but their utility in predicting the severity of odontogenic infections, particularly in the elderly, is not well established [18,19]. Given the complex interplay of factors influencing infection outcomes in older adults, there is a need to assess the predictive accuracy of these inflammatory scores in this specific context. Understanding their relevance can aid in early identification of high-risk patients and improve clinical outcomes.

Moreover, when using inflammatory scores in clinical practice, it is essential to understand the specific mechanisms and pathways through which odontogenic infections escalate in severity, particularly in the elderly. This demographic often exhibits a diminished immune response due to age-associated immunosenescence, which can delay the recognition of pathogens and slow the immune response, exacerbating the infection’s progression. The commonality and implications of dental caries, periodontitis and periapical abscesses as primary sources of OIs highlight the need for rigorous oral hygiene and regular dental check-ups in terms of preventing the initial onset and subsequent complications of these infections.

Furthermore, the Symptom Severity score and various inflammatory scores provide a quantifiable framework to gauge the infection’s impact. These scores incorporate not only clinical observations but also vital laboratory data, such as inflammatory markers and organ function tests, which are crucial in the elderly due to their often atypical presentation of symptoms. The predictive capacity of these scores could be particularly enhanced by integrating age-specific modifications that account for the altered physiological and immunological landscape in older patients. This approach could refine the thresholds or criteria within these scoring systems, improving their sensitivity and specificity for this age group. Such enhancements would support more tailored therapeutic strategies, allowing for early interventions that could mitigate the severe consequences of odontogenic infections in older adults.

This study aims to evaluate the effectiveness of APACHE II, CURB-65, SOFA, and NEWS2 scores in predicting the severity of odontogenic infections in elderly patients compared to younger patients. By identifying reliable predictors of severe OIs, clinicians can enhance patient management strategies, leading to improved outcomes and reduced morbidity and mortality in this vulnerable population.

## 2. Materials and Methods

### 2.1. Legal and Ethical Considerations

This retrospective cohort study was designed to evaluate the predictive accuracy of clinical scores APACHE II, CURB-65, SOFA, and NEWS2 in predicting severe COVID-19 outcomes in elderly patients aged 70 years and older. The study was conducted at the Department of Maxillofacial Surgery Department of City Emergency Hospital Timisoara (SCMUT), affiliated with the Victor Babes University of Medicine and Pharmacy, from Timisoara, Romania. Data were collected between 2020 until 2023. A control group of patients under 70 years old was included for comparative analysis. The study protocol was approved by the Institutional Review Board, and written informed consent was obtained from all participants or their legal guardians. The study adhered to the ethical standards of the Declaration of Helsinki, the EU Good Clinical Practice Directives (2005/28/EC) and the International Council for Harmonization of Technical Requirements for Pharmaceuticals for Human Use (ICH) guidelines. All patient data were anonymized to ensure confidentiality.

### 2.2. Patient Selection and Groups

A total of 194 patients diagnosed with odontogenic infections were included in the study. We included patients over the age of 18 with both clinical and radiological confirmation of odontogenic infections that necessitated hospitalization. Diagnostic criteria for these infections were detailed based on symptomatic presentations such as dental or facial pain, swelling, elevated body temperature and purulent discharge, corroborated by radiographic evidence indicating infection around dental structures. Exclusion criteria were clearly outlined to exclude individuals with non-odontogenic facial infections, those with immunodeficiency disorders or malignancies that could influence disease progression and those lacking comprehensive medical records, ensuring a homogeneous study population. The cohort was divided into two age-based groups: elderly patients aged 70 years and above and younger patients aged below 70 years, with an additional control group of healthy individuals without any history of odontogenic infections, matched by age and general health to the younger patients, serving as a baseline. Patient groups were defined as follows: elderly patients of age > 70 years (n = 49) and younger patients of age < 70 years (n = 64).

### 2.3. Data Collection and Clinical Assessments

Demographic data, medical history, comorbidities, clinical presentation, laboratory results and radiographic findings were extracted from electronic medical records. The Symptom Severity score (SS) for odontogenic infections is detailed as follows, with a maximum score of 20. For the Systemic Inflammatory Response Syndrome (SIRS) criteria, the scoring includes a temperature greater than 38.3 °C scores 1, a heart rate of over 90 bpm scores 1, a respiratory rate (RR) of 20/min or more scores 1, and a white blood cell (WBC) count below 4 or above 1.2 × 10^10^ scores 1, totaling a maximum of 4 points. Trismus is scored based on severity, with moderate trismus (less than 2 cm of mouth opening) scoring 3 points and severe trismus (less than 1 cm of mouth opening) scoring 4 points, also totaling a maximum of 4 points. Dysphagia is scored as mild when the patient has the ability to swallow most foods (earning 2 points), moderate when the patient has the inability to swallow fluids (scoring 4 points), and severe when the patient is drooling saliva (scoring 5 points, with a total maximum of 5 points). The presence of a collection in one facial space is scored based on severity: low severity (canine, vestibular) scores 1, moderate severity (buccal) scores 2, and high severity (all other spaces) scores 4, with a maximum of 5 points possible. A collection of two or more facial spaces automatically scores 5 points. Signs of dehydration, which include low blood pressure (BP), increased urea, or decreased skin turgor, score 1 point out of a possible 2. The presence of comorbidities such as diabetes mellitus, immunocompromised status, or chronic alcohol misuse also contributes 1 point. This scoring system helps in quantifying the severity of odontogenic infections based on clinical criteria (Table 1).

### 2.4. Inflammatory Scoring Systems

The inflammatory scores were calculated by attending physicians responsible for treating the patients included in the study. APACHE II (Acute Physiology and Chronic Health Evaluation II) is a system for classifying disease severity which estimates mortality risk based on 12 physiological variables, age, and pre-existing health conditions during the initial 24 h after ICU admission. CURB-65 is a clinical instrument that determines the severity of pneumonia and informs treatment choices using five criteria: confusion, urea levels, respiratory rate, blood pressure, and being 65 years or older. The SOFA (Sequential Organ Failure Assessment) score monitors a patient’s condition in the ICU and evaluates the degree and progression of organ failure through factors that include respiratory, coagulative, hepatic, cardiovascular, neurological, and renal functions. NEWS2 (National Early Warning Score 2) builds on its predecessor to better identify patient deterioration by consistently monitoring vital signs like respiratory rate, oxygen saturation, body temperature, systolic blood pressure, pulse rate and consciousness level.

### 2.5. Statistical Analysis

Data were analyzed using SPSS Statistics version 26.0 (IBM Corp., Armonk, NY, USA). Continuous variables were presented as means ± Standard Deviation (SD) and compared using a Student’s t-test or Mann–Whitney U test, as appropriate. Categorical variables were expressed as frequencies and percentages and compared using a Chi-square test or Fisher’s exact test. Receiver operating characteristic (ROC) curves were constructed to evaluate the predictive accuracy of the inflammatory scores, and the area under the curve (AUC) was calculated. Multivariate logistic regression analysis was performed to identify independent predictors of severe OI. *p*-values < 0.05 were considered statistically significant.

## 3. Results

### 3.1. Patient Demographics and Clinical Characteristics

The demographic and clinical characteristics of elderly patients (aged over 70 years, n = 49) and younger controls (under 70 years, n = 64) were analyzed. The average age of elderly patients was significantly higher than that of controls (74.58 ± 3.29 vs. 52.37 ± 10.54, *p* < 0.001). There were no significant differences in gender distribution between the two groups (57.14% male in elderly vs. 59.38% in controls, *p* = 0.823). Body Mass Index (BMI) was significantly lower in the elderly group compared to the controls (25.13 ± 4.08 vs. 27.86 ± 5.12, *p* = 0.001). The prevalence of smoking and alcohol use was lower in the elderly, although these differences were not statistically significant (*p* = 0.085 and *p* = 0.070, respectively). Notably, comorbid conditions such as diabetes mellitus (38.78% vs. 12.50%, *p* < 0.001), cardiovascular disease (44.90% vs. 15.63%, *p* < 0.001), and chronic kidney disease (22.45% vs. 7.81%, *p* = 0.025) were significantly more prevalent among the elderly, indicating a higher burden of health issues in this population (Table 2).

### 3.2. Symptom Severity (SS) Score Comparison

In the comparative analysis of the Symptom Severity score components for patients with odontogenic infections from Table 3, significant differences were observed between elderly patients (over 70 years, n = 49) and younger controls (under 70 years, n = 64). The Systemic Inflammatory Response Syndrome (SIRS) criteria score was significantly higher in the elderly group (2.85 ± 0.73) compared to the controls (1.92 ± 0.68, *p* < 0.001). Similarly, elderly patients had a higher trismus score (2.67 ± 1.01 vs. 1.89 ± 0.94, *p* < 0.001) and dysphagia score (3.17 ± 1.20 vs. 2.05 ± 0.82, *p* < 0.001), indicating more severe manifestations of these symptoms. Fascial space involvement, which reflects the extent of infection, was also more pronounced in the elderly (3.45 ± 0.98 vs. 2.01 ± 0.76, *p* < 0.001). Additionally, scores reflecting dehydration and comorbid conditions were significantly higher in the elderly group (0.40 ± 0.49 vs. 0.10 ± 0.33, *p* = 0.002), underscoring the increased vulnerability of this population in the context of odontogenic infections.

### 3.3. Inflammatory Scores at Admission

In the study assessing inflammatory scores at admission among patients with odontogenic infections (Table 4), significant differences were found between elderly patients (over 70 years, n = 49) and younger controls (under 70 years, n = 64). The APACHE II score, which evaluates the severity of disease and likelihood of mortality, was significantly higher in elderly patients (19.75 ± 5.12) compared to controls (14.82 ± 4.63, *p* < 0.001). Similarly, the CURB-65 score, used to assess pneumonia severity, was higher among the elderly (3.07 ± 0.89 vs. 1.45 ± 0.67, *p* < 0.001). The SOFA score, which tracks organ failure progression, was also considerably higher in the elderly group (5.47 ± 2.18 vs. 2.64 ± 1.32, *p* < 0.001). Additionally, the NEWS2 score, a measure of early warning signs for clinical deterioration, was notably higher in elderly patients (6.85 ± 2.31 vs. 3.56 ± 1.39, *p* < 0.001).

### 3.4. Predictive Accuracy of Inflammatory Scores

The APACHE II score demonstrated an AUC of 0.83, with an optimal cutoff of 17.5, yielding a sensitivity of 82.14% and a specificity of 78.95% (*p* < 0.001). The CURB-65 score had an AUC of 0.81 with a cutoff of 2.5, achieving a sensitivity of 85.71% and a specificity of 73.80% (*p* < 0.001). The SOFA score showed superior performance with an AUC of 0.89, a cutoff of 4.5, a sensitivity of 88.89%, and a specificity of 84.62% (*p* < 0.001). Lastly, the NEWS2 score, with an AUC of 0.78 and a cutoff of 5.5, had a sensitivity of 78.57% and a specificity of 75.30% (*p* < 0.001), as seen in Table 5.

The incidence of sepsis was significantly higher in elderly patients, at 22.45% compared to 7.81% in controls (*p* = 0.035). Similarly, ICU admissions were more frequent among the elderly, with 18.37% being admitted compared to only 6.25% of younger patients (*p* = 0.048). Additionally, the average length of hospital stays was significantly longer for the elderly group, averaging 12.45 days versus 7.62 days for the controls (*p* < 0.001). Although not statistically significant, mortality rates were higher among the elderly at 6.12% compared to 1.56% in younger patients (*p* = 0.208), as presented in Table 6.

The analysis revealed that an age greater than 70 years significantly increased the odds of severe infections, with an odds ratio (OR) of 2.85 and a 95% confidence interval (CI) ranging from 1.32 to 6.17 (*p* = 0.007). An APACHE II score greater than 17.5 also indicated a higher risk, with an OR of 2.12 and a 95% CI from 1.15 to 3.90 (*p* = 0.016). A SOFA score greater than 4.5 showed the strongest association, with an OR of 3.45 and a 95% CI from 1.82 to 6.55 (*p* < 0.001). Additionally, having diabetes mellitus was associated with an OR of 2.76 (95% CI: 1.32–5.71, *p* = 0.007), while cardiovascular disease was associated with an OR of 2.48 (95% CI: 1.21–5.08, *p* = 0.013), as seen in Table 7.

## 4. Discussion

### 4.1. Analysis of Findings

Our findings demonstrate that elderly patients (>70 years) have significantly higher Symptom Severity scores and inflammatory scores at admission, indicating more severe infections and worse clinical outcomes. The elevated SS scores among elderly patients can be attributed to age-related physiological changes, decreased immune responses and a higher prevalence of comorbidities. Among the inflammatory scores evaluated, the SOFA score demonstrated the highest predictive accuracy for severe OIs in elderly patients, with an AUC of 0.89, and it also had the highest sensitivity (88.8%) and specificity (84.6%). This suggests that organ dysfunction, as assessed by the SOFA score, is a critical factor in determining the severity of odontogenic infections in this population. The APACHE II and CURB-65 scores also showed good predictive values, reinforcing their utility in clinical assessment.

The study findings highlight the significant correlation between comorbidities in elderly patients and the increased severity of odontogenic infections, underscoring the predictive power of these comorbid conditions. Elderly individuals demonstrate a higher prevalence of diabetes, cardiovascular diseases, and chronic kidney disease, which are associated with more severe symptoms and poorer clinical outcomes. This correlation suggests that the presence of such comorbidities could serve as vital indicators for heightened surveillance and proactive therapeutic interventions. By identifying these high-risk factors, healthcare providers can potentially establish tailored therapeutic measures aimed at minimizing the risk of severe infections and improving the management of odontogenic infections in elderly patients. Moreover, the elevated inflammatory scores observed in elderly patients, as highlighted by the study, underscore a distinct age-related modulation of the immune response to odontogenic infections. Aging is associated with immunosenescence, which alters both the innate and adaptive immune systems, leading to a generally pro-inflammatory state known as “inflammaging.” This condition can exacerbate the body’s response to infections, making elderly individuals more susceptible to severe manifestations of odontogenic infections. Our findings align with this theory, as higher SOFA scores in older adults likely reflect this compounded immune response. Furthermore, comparing these age-related differences in inflammatory responses with the existing literature reveals a consistent pattern where older patients typically exhibit more pronounced inflammatory markers.

Regarding outcomes among patients with OIs, Flynn et al. [20] prospectively assessed 37 hospitalized patients, revealing that trismus and dysphagia were common symptoms, occurring in over 70% of cases, and highlighted a high rate of PCN-resistant organisms at 19%, contributing to a 21% PCN therapeutic failure rate. This study underscored the necessity of considering alternative antibiotics like clindamycin due to high resistance rates and emphasized the frequent involvement of the masticator space in severe OI. Conversely, Neal et al. [21] focused on the relationship between the odontogenic infection severity score (OISS) and the likelihood of difficult intubations in surgical settings. Their retrospective cohort study identified that patients with an OISS ≥ 5 were nearly four times more likely to experience difficult intubations compared to those with lower scores, providing a clear statistical link with a sensitivity of 69% and a specificity of 63%.

In examining the diagnostic utility of hematologic and inflammatory markers for odontogenic infections, Kusumoto et al. [22] and Hammad et al. [23] contributed significant findings. Kusumoto et al.‘s retrospective analysis revealed that severe cases such as deep neck abscesses and necrotizing soft tissue infections (NSTIs) could be differentiated using specific markers, with the Laboratory Risk Indicator for Necrotizing Fasciitis (LRINEC) score showing a cutoff value of 6 and a CRP + neutrophil-to-lymphocyte ratio (NLR) with a cutoff of 27 effectively identifying NSTI cases. Their decision tree analysis demonstrated an 89.3% accuracy using the systemic immune–inflammation index (SII) for categorizing severe infections. Conversely, Hammad et al. focused on correlating serum markers with odontogenic infection severity, finding that serum glucose and white blood cell (WBC) counts were significantly higher in more severe infections, with mean values reaching statistical significance (*p* = 0.001 and *p* = 0.036, respectively), while CRP and body temperature did not show significant differences.

Similarly, Ghasemi et al. [24] conducted a systematic review to assess the role of the neutrophil to lymphocyte ratio (NLR) as a biomarker in OIs, finding that an elevated NLR was significantly correlated with disease severity, prolonged hospital stays, and the need for postoperative antibiotics, with the optimal NLR cutoff being identified as 5.19, which predicted disease severity with a specificity of 81% and a sensitivity of 51%. Furthermore, the study highlighted the statistical significance of NLR in predicting mortality in OIs patients (*p* = 0.026, specificity of 55.5%, sensitivity of 70.8%). In a similar manner, the study by Pham Dang et al. [25] identified key predictors of complex management in severe OI cases such as penicillin allergies, psychiatric disorders, oropharyngeal edemas, and fever. Their findings emphasized the need for tailored management strategies based on these predictors to effectively address the complications associated with severe OIs, further underlining the utility of measurable parameters in enhancing clinical outcomes.

Moreover, Mirochnik et al. [26] assessed 100 cases and determined that severity scoring significantly predicted the length of hospital stay (LOS), with “Moderate & Severe” cases having a longer median LOS of 4.5 days compared to 3 days for “Low” severity cases, highlighting the practical application of severity scores in clinical decision-making (*p* = 0.00016). In a similar manner, Aditya et al. [27], through a retrospective analysis of 259 patients, identified multiple factors that significantly influenced the prognosis of odontogenic infections. They found that the severity of the infection, the number of spaces involved, trismus, dysphagia, and dyspnea were critical in predicting both the length of hospital stay and the necessity for re-exploration, with severity and the number of spaces affected both showing highly significant impacts (*p* < 0.001).

These findings align with previous research indicating that elderly patients are at higher risk of severe infections and complications. The use of inflammatory scores can aid clinicians in early identification of high-risk patients, allowing for timely interventions and potentially improved outcomes.

### 4.2. Study Limitations

The limitations of this study include its retrospective design and the potential for selection bias. Additionally, the sample size, particularly for mortality outcomes, may limit the generalizability of the results. Future prospective studies with larger sample sizes are needed to validate these findings.

## 5. Conclusions

This study demonstrates that inflammatory scores, particularly the SOFA score, are effective in predicting the severity of odontogenic infections in elderly patients. Elderly patients present with more severe infections and worse clinical outcomes compared to younger patients. The integration of inflammatory scoring systems into clinical practice can enhance the early identification of high-risk patients, allowing for prompt and targeted management strategies to improve patient outcomes in this vulnerable population. Future research should focus on refining these scoring systems to increase their specificity and sensitivity for elderly populations. Studies could explore the incorporation of additional biomarkers or clinical parameters that reflect age-specific responses to infection. Moreover, longitudinal studies are necessary to evaluate the long-term effects of early intervention based on inflammatory scores and to assess the cost-effectiveness of such strategies in routine healthcare settings. These efforts will further our understanding of the dynamics of odontogenic infections in the elderly and help to optimize therapeutic approaches tailored to this group’s unique needs.

## Figures and Tables

**Table 1:** The Symptom Severity score (SS) of odontogenic infections.

Criteria		Score	Max Score
Systemic Inflammatory Response Syndrome (SIRS)	Temperature > 38.3 °C	1	4
	Heart rate > 90 bpm	1	
	RR 20/min	1	
	WBC < 4 or >1.2 × 10^10^	1	
Trismus	Moderate < 2 cm	3	4
	Severe < 1 cm	4	
Dysphagia	Mild—able to swallow most foods	2	5
	Moderate—unable to swallow fluids	4	
	Severe—drooling saliva	5	
Collection in 1 fascial space	Low severity (canine, vestibular)	1	5
	Moderate severity (buccal)	2	
	High severity (all other spaces)	4	
Collection in 2 or more fascial spaces		5	
Sign of dehydration (↓ BP/↑ Urea/↓ Skin turgor)		1	2
Comorbidities: diabetes mellitus, immunocompromised status, known or suspected chronic alcohol misuser		1	
Total Score			20

SIRS—Systemic Inflammatory Response Syndrome; BP—blood pressure; RR—respiratory rate; WBC—white blood cells; ↓—decrease; ↑—increase.

**Table 2:** Demographic and Clinical Characteristics of Elderly and Younger Patients (<70 years old) with Odontogenic Infections.

Variables	Patients > 70 Years (n = 49)	Patients < 70 Years (n = 64)	*p*
Age (years, mean ± SD)	74.58 ± 3.29	52.37 ± 10.54	<0.001
Gender (Male, n (%))	28 (57.14%)	38 (59.38%)	0.823
BMI (kg/m^2^, mean ± SD)	25.13 ± 4.08	27.86 ± 5.12	0.001
Smoking (n (%))	7 (14.29%)	18 (28.13%)	0.085
Alcohol Use (n (%))	5 (10.20%)	15 (23.44%)	0.070
Comorbidities (n (%))			0.865
Diabetes Mellitus	19 (38.78%)	8 (12.50%)	<0.001
Cardiovascular Disease	22 (44.90%)	10 (15.63%)	<0.001
Chronic Kidney Disease	11 (22.45%)	5 (7.81%)	0.025

BMI—Body Mass Index; SD—Standard Deviation.

**Table 3:** Comparison of Symptom Severity Score Components in Patients with Odontogenic Infections.

Variables (Mean ± SD)	Patients > 70 Years (n = 49)	Patients < 70 Years (n = 64)	*p*
SIRS Criteria score	2.85 ± 0.73	1.92 ± 0.68	<0.001
Trismus score	2.67 ± 1.01	1.89 ± 0.94	<0.001
Dysphagia score	3.17 ± 1.20	2.05 ± 0.82	<0.001
Fascial Space Involvement score	3.45 ± 0.98	2.01 ± 0.76	<0.001
Dehydration/Comorbidity score	0.40 ± 0.49	0.10 ± 0.33	0.002

SD—Standard Deviation; SIRS—Systemic Inflammatory Response Syndrome.

**Table 4:** Inflammatory Scores at Admission Among Patients with Odontogenic Infections.

Variables (Mean ± SD)	Patients > 70 Years (n = 49)	Patients < 70 Years (n = 64)	*p*
APACHE II	19.75 ± 5.12	14.82 ± 4.63	<0.001
CURB-65	3.07 ± 0.89	1.45 ± 0.67	<0.001
SOFA	5.47 ± 2.18	2.64 ± 1.32	<0.001
NEWS2	6.85 ± 2.31	3.56 ± 1.39	<0.001

SD—Standard Deviation; APACHE II—Acute Physiology and Chronic Health Evaluation II; CURB-65—confusion, urea, respiratory rate, blood pressure, age ≥ 65; SOFA—Sequential Organ Failure Assessment; NEWS2—National Early Warning Score 2.

**Table 5:** Inflammatory Scores at Admission Among Patients with Odontogenic Infections.

Score	AUC	Optimal Cutoff	Sensitivity (%)	Specificity (%)	*p*
APACHE II	0.83	17.5	82.14	78.95	<0.001
CURB-65	0.81	2.5	85.71	73.8	<0.001
SOFA	0.89	4.5	88.89	84.62	<0.001
NEWS2	0.78	5.5	78.57	75.3	<0.001

APACHE II—Acute Physiology and Chronic Health Evaluation II; CURB-65—confusion, urea, respiratory rate, blood pressure, age ≥ 65; SOFA—Sequential Organ Failure Assessment; NEWS2—National Early Warning Score 2; AUC—area under curve.

**Table 6:** Clinical Outcomes Among Patients with Odontogenic Infections.

Outcome	Patients > 70 Years (n = 49)	Patients < 70 Years (n = 64)	*p*
Sepsis (n (%))	11 (22.45%)	5 (7.81%)	0.035
ICU Admission (n (%))	9 (18.37%)	4 (6.25%)	0.048
Length of Hospital Stay (days)	12.45 ± 3.78	7.62 ± 2.54	<0.001
Mortality (n (%))	3 (6.12%)	1 (1.56%)	0.208

ICU—Intensive Care Unit.

**Table 7:** Multivariate Logistic Regression Analysis for Predictors of Severe Odontogenic Infections.

Variable	Odds Ratio (OR)	95% Confidence Interval	*p*
Age (>70 years)	2.85	1.32–6.17	0.007
APACHE II (>17.5)	2.12	1.15–3.90	0.016
SOFA (>4.5)	3.45	1.82–6.55	<0.001
Diabetes Mellitus	2.76	1.32–5.71	0.007
Cardiovascular Disease	2.48	1.21–5.08	0.013

APACHE II—Acute Physiology and Chronic Health Evaluation II; SOFA—Sequential Organ Failure Assessment.

## Data Availability

The data presented in this study are available on request from the corresponding author.
